# Mr. Pearson on Vaccination in Canton

**Published:** 1811-12

**Authors:** A. Pearson

**Affiliations:** Canton


*To the Editors of the Medical and Physical Journal.*

Gentlemen,

FROM your insertion in p. 488 of your 18th vol. of a communication of mine to Sir George Staunton, which was submitted to you by Dr. Maton, respecting the introduction and progress of Vaccination in China, I have been induced to present to you a report on the same subject in continuation, as an article of intelligence likely to partake of the interest which the Doctor is pleased to ascribe to the notice alluded to, with some of your readers.

It is merely to state, that the practice has been carried on, to this date, here and at Macao, extensively enough during the ravages of the small-pox, (the visits of which are in this country annual); but as I anticipated in the communication in question, has been much slighted when the danger went past, so as to render the preservation of it difficult: indeed from August, 1810, to January, 1811, we were entirely without it at both places; and when, in consequence of the reappearance of the small-pox, applicants for vaccination became numerous, I had the satisfaction to find that it had been kept up in a district at some distance from hence, by a Chinese whom I had instructed, and of receiving from that quarter a fresh stock, from which the practice is now amply kept up here, and has been revived at Macao.

I may add, however, that the estimate of the chances against its preservation, which I then offered, is not much less applicable at the present time, although confidence in its efficacy seems to increase; and I believe what I mentioned as a great desideratum, that some of the natives would prosecute the practice from views of emolument, will this season be supplied in one or two instances.

I have the honour to remain,

Your most obedient Servant,

A. PEARSON.

*Canton, March* 23, 1811.

